# Rotavirus P[8] Infections in Persons with Secretor and Nonsecretor Phenotypes, Tunisia

**DOI:** 10.3201/eid2111.141901

**Published:** 2015-11

**Authors:** Siwar Ayouni, Khira Sdiri-Loulizi, Alexis de Rougemont, Marie Estienney, Katia Ambert-Balay, Serge Aho, Sabeur Hamami, Mahjoub Aouni, Mohamed Neji-Guediche, Pierre Pothier, Gaël Belliot

**Affiliations:** National Reference Center for Enteric Viruses, Dijon, France (S. Ayouni, A. de Rougemont, M. Estienney, K. Ambert-Balay, P. Pothier, G. Belliot);; Monastir University, Monastir, Tunisia (S. Ayouni, K. Sdiri-Loulizi, M. Aouni);; Public Hospital of Dijon, Dijon (S. Aho);; Hospital Fattouma Bourguiba, Monastir (S. Hamami, M. Neji-Guediche)

**Keywords:** rotavirus, rotavirus P[8], human blood group antigens, HBGA, nonsecretor, secretor, gastroenteritis, children, viruses, Tunisia, Lewis antigen

## Abstract

To determine whether rotavirus infections are linked to secretor status, we studied samples from children in Tunisia with gastroenteritis. We phenotyped saliva for human blood group antigens and tested feces for rotavirus. Rotavirus was detected in 32/114 patients. Secretor genotyping showed that P[8] rotavirus infected secretors and nonsecretors, and infection correlated with presence of Lewis antigen.

Each year, millions of persons worldwide suffer from acute gastroenteritis. Group A rotavirus is the leading cause of acute gastroenteritis in children <5 years of age. The disease causes ≈453,000 deaths annually, mostly in developing countries (*1*); however, the number of cases has declined in industrialized countries where vaccines have been recommended (*2*).

Recent findings showed that human blood group antigens (HBGAs) might be involved in rotavirus attachment to intestinal cells (*3**,**4**,**5**,**6*). Expression of the HBGAs (A, B, H, and Lewis antigens) in saliva and on the surface of intestinal cells is driven by the *FUT2* (A, B, and H antigens [secretor]) and *FUT3* (Lewis antigens) genes, which express type 2 and type 3 fucosyltransferases, respectively. Approximately 20% of the white population is homozygous for a recessive point mutation of the *FUT2* gene, which leads to the absence of A, B, and H antigen expression, also called the nonsecretor phenotype. There is also a Lewis-negative phenotype resulting from various mutations of the *FUT3* gene (*7*).

The entry of rotavirus into cells involves several factors. Human and porcine rotaviruses could specifically interact with H antigen type 1, Lewis b antigen, or Lewis a antigen through their viral protein (VP) 8 and VP5 during the attachment phase (*3*,*4*,*8*). Of note, the HBGA binding profile is P genotype–dependent (*4*), and rotavirus infection correlates with the secretor and partial secretor phenotype (i.e., with active *FUT2* gene status) (*5*,*6*,*9*). However, in some studies, no association has been observed between HBGAs from blood cells (*10*), including Lewis antigens (*11*), and rotavirus infection. A recent epidemiologic survey of children in the region of Monastir, Tunisia, gave us the opportunity to determine whether rotavirus infections in children could be linked to secretor status and HBGAs.

## The Study

During November 2011–February 2012, feces and saliva samples were collected from 114 children <6 years of age who were seen for acute gastroenteritis at the Fattouma Bourguiba children’s hospital (Monastir). For 98 of these patients, blood samples were also collected at symptom onset for *FUT2* genotyping by sequencing for the A385T and G428A nonsense mutations from total blood DNA (*9*). The study was approved by the Ethics Committee of the Fattouma Bourguiba University Hospital in Monastir, and informed consent was obtained from the parents of the 114 study participants.

The feces were first screened for the presence of group A rotavirus antigen by using the Premier Rotaclone detection kit (Meridian Bioscience, Inc., Paris, France). The remainder of the suspension was used for the extraction of nucleic acids by using a Nuclisens easyMAG system (bioMérieux, Marcy l’Étoile, France) according to the manufacturer’s instructions. RNA was eluted in a final volume of 110 μL and used for the molecular detection and typing of rotavirus. Norovirus PCR detection is described elsewhere (*12*).

Samples positive for rotavirus by ELISA were further confirmed and genotyped by PCR as described previously (Technical Appendix Table). Of the 114 patients, 32 had confirmed rotavirus infections by ELISA and PCR. Of the 32 confirmed cases, 24 (75%) occurred during the cold season, and 26 (80%) occurred in children <14 months of age; the mean age for infected persons was 8.1 months. We used ELISAs to screen the saliva of rotavirus-positive patients for A and B antigens (anti-A and anti-B mouse IgG from DIAGAST, Loos, France), H antigen (anti-H specific IgM from Thermo Fisher Scientific, Villebon sur Yvette, France), and Lewis antigens (anti-Lewis a [clone 7LE] and anti-Lewis b [clone 2–25LE] hybridoma supernatants; gift from Jacques Bara, INSERM U673). Among the secretor phenotype–positive rotavirus patients, no blood group antigen nor P or G genotypes (in feces specimens) were significantly overrepresented. For comparison, we assessed the distribution of ABO blood groups and Lewis antigens among patients with norovirus and rotavirus; no statistical difference was found (Table 1). Rotavirus infection was observed only in Lewis antigen–positive patients (p = 0.017, exact logistic regression); however, the prevalence of the Lewis antigen–negative phenotype in the population was low.

**Table 1:** Distribution of rotavirus and norovirus cases among 114 children <6 years of age by ABO blood group, Lewis phenotype status, and nonsecretor status, Tunisia, November 2011–February 2012*

Phenotype	No. rotavirus-positive patients	No. norovirus-positive patients
ABO and Lewis antigen distribution in patients, n = 90		
O		
Le+, n = 37	12	21
Le−, n = 1	0	0
A		
Le+, n = 26	10	7
Le−, n = 1	0	0
B		
Le+, n = 16	4	4
Le−, n = 3	0	2
AB		
Le+, n = 6	2	1
Le−, n = 0	0	0
Nonsecretor group, Lewis antigen status, n = 24		
Positive, n = 24	4	7
Negative, n = 0	0	0
Total	32	42

*All children had gastroenteritis. Paired saliva and feces specimens were collected from each patient (N = 114) and screened for the presence of ABO and Lewis antigens (saliva) and rotavirus and norovirus (feces). Le+, positive for Lewis antigen; Le−, negative for Lewis antigen.

Among the 32 rotavirus isolates, 30 were genotype P[8] and 2 were genotype P[4] (Table 2). G9, G3, and G1 were the most common genotypes and were detected in 13 (40%), 8 (25%), and 7 (21%) of the cases, respectively. The G genotype could not be determined for 1 P[8] genotype isolate. Genotypes G1, G3, G4, and G9 were all associated with the P[8] genotype, and genotype G2 was associated with the P[4] genotype. Rotavirus G9P[8] strains were predominant (n = 12), followed by G3P[8] (n = 8) and G1P[8] (n = 7) strains.

**Table 2:** Distribution of ABO blood groups and Lewis antigens among 32 children <6 years of age infected with various rotavirus strains in Monastir, Tunisia, November 2011–February 2012*

Isolated rotavirus strain, patient *FUT2 *genotype	No. patients by ABO blood group and Lewis antigen status†												No. nonsecretor patients	
	O			A			B			AB				
	Le+	Le−		Le+	Le−		Le+	Le−		Le+	Le−		Le+	Le−
G9P[8]	4	0		1	0		2	0		2	0		3	0
Se/Se	0	0		1	0		1	0		1	0		0	0
Se/se	4	0		0	0		1	0		1	0		0	0
se/se	0	0		0	0		0	0		0	0		3	0
G3P[8]	1	0		4	0		2	0		0	0		1	0
Se/Se	0	0		2	0		1	0		0	0		0	0
Se/se	1	0		1	0		1	0		0	0		0	0
se/se	0	0		0	0		0	0		0	0		1	0
G1P[8]	4	0		3	0		0	0		0	0		0	0
Se/Se	0	0		0	0		0	0		0	0		0	0
Se/se	4	0		2	0		0	0		0	0		0	0
G4P[8]	1	0		1	0		0	0		0	0		0	0
Se/se	0	0		1	0		0	0		0	0		0	0
G2P[4]	2	0		0	0		0	0		0	0		0	0
Se/Se	1	0		0	0		0	0		0	0		0	0
P[8]	0	0		1	0		0	0		0	0		0	0
Se/Se	0	0		1	0		0	0		0	0		0	0
Total	12	0		10	0		4	0		2	0		4	0
Se/Se	1	0		4	0		2	0		1	0		0	0
Se/se	9	0		4	0		2	0		1	0		0	0
se/se	0	0		0	0		0	0		0	0		4	0

*Le+, positive for Lewis antigen; Le−, negative for Lewis antigen; Se/Se, homozygous secretor; Se/se, heterozygous secretor; se/se, nonsecretor. †Human blood group antigen distribution was determined by using a typing assay with saliva samples.

For 3 G9P[8] and 1 G3P[8] rotavirus-positive patients, saliva samples were negative for Lewis b antigen (mean absorbance at 450 nm was 0.25) and positive for the presence of Lewis a antigen (mean absorbance at 450 nm was 3.67), suggesting that the patients were Lewis-positive and nonsecretors. A total of 98 blood samples were genotyped by sequencing of the *FUT2* gene. Homozygous secretor, heterozygous secretor, and nonsecretor genotypes represented 23.47%, 54.08%, and 22.45% of the cohort, respectively. All nonsecretors (n = 22) harbored the G428A mutation. The A385T mutation was absent. Blood samples were available for 28 of the 32 rotavirus-positive patients. Of these 28 patients, 24 were homozygous and heterozygous secretors and 4 were nonsecretors. Because rotaviruses were P- and G-typed by PCR using VP4- and VP7-specific primers, we further confirmed the presence of rotavirus in samples from nonsecretor patients. For 3 of the samples, we used a TaqMan-based quantitative reverse transcription PCR with VP2-specific primers to detect rotavirus (*13*), and for 2 rotavirus isolates (GenBank accession nos. KP862856 and KP862857) for which feces samples were still available, we confirmed the P[8] genotype by sequencing.

## Conclusions

Our findings show that rotaviruses can infect secretor and nonsecretor Lewis antigen–positive persons, which suggests that rotavirus infection is not associated with the secretor phenotype or HBGA type. However, it should be noted that one limitation of our study was the small size of our cohort. A larger number of cases might provide new insights about the affinity of rotavirus toward certain types of HBGAs; a larger study with a more robust statistical analysis might confirm that rotavirus infections only occur in Lewis antigen–positive persons. In addition, we detected genotype P[8] rotavirus infection in both secretor and nonsecretor patients; this finding was not observed in previous studies (*5*,*6*,*9*). P[8] infection of nonsecretors might be associated with preexisting health conditions, and healthy nonsecretors might never be infected by P[8] rotavirus.

We and others (*3**,**4*,^6^) have characterized the secretor and Lewis phenotypes related to infection by rotaviruses. The interaction between rotavirus particles and HBGAs might constitute the first step in the attachment to the cell before internalization of the virus particle, after binding with integrins (*4*,^5^). However, other types of ligand, such as non-HBGA ligands and bacteria from intestinal flora, might also play a role during the infection process, as recently shown for noroviruses (*14*,*15*).

In conclusion, our data and that of others show that rotavirus infection might be correlated with genetic factors, such as HBGAs. Further studies will be required to determine the exact role of HBGA ligands and other ligands in rotavirus infection.

Technical Appendix Summary of primers used for rotavirus typing.
