# Supplementary material for: Rotavirus P[8] Infections in Persons with Secretor and Nonsecretor Phenotypes, Tunisia
Source: Emerg Infect Dis. 2015 Nov;21(11):2055–8. doi: 10.3201/eid2111.141901 (PMC4622234; doi:10.3201/eid2111.141901)
Supplement: Technical Appendix — Summary of primers used for rotavirus typing. [file 14-1901-Techapp-s1.pdf]

Article DOI: http://dx.doi.org/10.3201/eid2111.141901

Rotavirus P[8] Infections in Persons with Secretor and Nonsecretor Phenotypes, Tunisia

Technical Appendix

Technical Appendix Table. Summary of the previously designed primers used for rotavirus typing in a study of children <6 years of age with secretor and nonsecretor phenotypes, Monastir, Tunisia, November 2011–February 2012

Primer	Sequence	Polarity	Primer location	Reference
VP7-F	5′-ATGTATGGTATTGAATATACCAC-3′	+	49–71	(*1*)
VP7-R	5′-AACTTGCCACCATTTTTTCC-3′	-	914–933	(*1*,*2*)
VP4-F	5′-TATGCTCCAGTNAATTGG-3′	+	132–149	(*3*)
VP4-R	5′-ATTGCATTTCTTTCCATAATG-3′	-	775–795	(*3*)
VP6-F	5′-GACGGVGCRACTACATGGT-3′	+	747–766	(*4*)
VP6-R	5′-GTCCAATTCATNCCTGGTGG-3′	-	1106–1126	(*4*)
aBT1	5′-CAAGTACTCAAATCAATGATGG-3′	+	314–335	(*5*)
aCT2	5′-CAATGATATTAACACATTTTCTGTG-3′	+	411–435	(*5*)
G3	5′-ACGAACTCAACACGAGAGG-3′	+	250–269	(*2*)
aDT4	5′-CGTTTCTGGTGAGGAGTTG-3′	+	480–498	(*5*)
aAT8	5′-GTCACACCATTTGTAAATTCG-3′	+	178–198	(*5*)
G9	5′-CTTGATGTGACTAYAAATAC-3′	+	757–776	(*2*,*5*)
G10	5′-ATGTCAGACTACARATACTGG-3′	+	666–687	(*2*)
2T-1	5′-CTATTGTTAGAGGTTAGAGTC-3′	-	474–494	(*6*)
3T-1	5′-TGTTGATTAGTTGGATTCAA-3′	-	259–278	(*6*)
1T-1D	5′-TCTACTGGRTTRACNTGC-3′	-	339–356	(*7*)
4T-1	5′-TGAGACATGCAATTGGAC-3′	-	385–402	(*6*)
5T-1	5′-ATCATAGTTAGTAGTCGG-3′	-	575–594	(*6*)
P[11]	5′-GTAAACATCCAGAATGTG-3′	-	305–323	(*2*)

References

1. Gómara MI, Cubitt D, Desselberger U, Gray J. Amino acid substitution within the VP7 protein of G2 rotavirus strains associated with failure to serotype. J Clin Microbiol. 2001;39:3796–8. PubMed http://dx.doi.org/10.1128/JCM.39.10.3796-3798.2001

2. Iturriza-Gómara M, Kang G, Gray J. Rotavirus genotyping: keeping up with an evolving population of human rotaviruses. J Clin Virol. 2004;31:259–65. PubMed http://dx.doi.org/10.1016/j.jcv.2004.04.009

3. Simmonds MK, Armah G, Asmah R, Banerjee I, Damanka S, Esona M, et al. New oligonucleotide primers for P-typing of rotavirus strains: Strategies for typing previously untypeable strains. J Clin Virol. 2008;42:368–73. PubMed http://dx.doi.org/10.1016/j.jcv.2008.02.011

4. Iturriza Gómara M, Wong C, Blome S, Desselberger U, Gray J. Molecular characterization of VP6 genes of human rotavirus isolates: correlation of genogroups with subgroups and evidence of independent segregation. J Virol. 2002;76:6596–601. PubMed http://dx.doi.org/10.1128/JVI.76.13.6596-6601.2002

5. Gouvea V, Glass RI, Woods P, Taniguchi K, Clark HF, Forrester B, et al. Polymerase chain reaction amplification and typing of rotavirus nucleic acid from stool specimens. J Clin Microbiol. 1990;28:276–82. PubMed</jrn>

6. Gentsch JR, Glass RI, Woods P, Gouvea V, Gorziglia M, Flores J, et al. Identification of group A rotavirus gene 4 types by polymerase chain reaction. J Clin Microbiol. 1992;30:1365–73. PubMed</jrn>

7. Iturriza-Gómara M, Green J, Brown DW, Desselberger U, Gray JJ. Diversity within the VP4 gene of rotavirus P[8] strains: implications for reverse transcription-PCR genotyping. J Clin Microbiol. 2000;38:898–901. PubMed
